# Clinical and mutational spectrum of paediatric Charcot-Marie-Tooth disease in a large cohort of Chinese patients

**DOI:** 10.3389/fgene.2023.1188361

**Published:** 2023-07-13

**Authors:** Yan Ma, Xiaohui Duan, Xiaoxuan Liu, Dongsheng Fan

**Affiliations:** ^1^ Department of Neurology, Peking University Third Hospital, Beijing, China; ^2^ Beijing Key Laboratory of Biomarker and Translational Research in Neurodegenerative Diseases, Beijing, China; ^3^ Key Laboratory for Neuroscience, National Health Commission/Ministry of Education, Peking University, Beijing, China; ^4^ China-Japan Friendship Hospital, Beijng, China

**Keywords:** paediatric Charcot-Marie-Tooth disease, inherited peripheral neuropathy, genetic distribution, genotype-phenotype correlations, *de novo* variants

## Abstract

**Background:** Charcot-Marie-Tooth disease (CMT) is the most common inherited neurological disorder suffered in childhood. To date, the disease features have not been extensively characterized in the Chinese paediatric population. In this study, we aimed to analyse the clinical profiles and genetic distributions of a paediatric CMT cohort in China.

**Methods:** A total of 181 paediatric CMT patients were enrolled. After preexcluding *PMP22* duplication/deletion by multiplex ligation-dependent probe amplification (MLPA), Sanger sequencing, targeted next-generation sequencing (NGS) or whole-exome sequencing (WES) was performed to obtain a genetic diagnosis. Detailed information was collected to explore the spectrum of subtypes and genotype-phenotype correlations.

**Results:** Pathogenic mutations were identified in 68% of patients in this study; with *PMP22* duplication, MFN2 and *GJB1* were the most frequent disease-causing genes. Of note, respect to the higher prevalence worldwide, CMT1A (18.2%) was relatively lower in our cohort. Besides, the mean age at onset (8.3 ± 5.7 years) was significantly older in our series. In genotype-phenotype analyse, *PMP22* point mutations were considered the most severe genotypes and were mostly *de novo*. In addition, the *de novo* mutations were identified in up to 12.7% of all patients, which was higher than that in other studies.

**Conclusion:** We identified a relatively lower detection rate of *PMP22* duplication and a higher frequency of *de novo* variants among paediatric patients in China. We also identified the genetic and phenotypic heterogeneity of this cohort, which may provide clues for clinicians in directing genetic testing strategies for Chinese patients with early-onset CMT.

## Introduction

Charcot-Marie-Tooth disease (CMT) is one of the most common inherited peripheral neuropathies with a prevalence of 1 in 2,500 individuals (Skre, 1974). As a collection of clinically and genetically heterogeneous disorders, CMT varies strikingly in terms of phenotypes and severity, especially in paediatric populations. In clinical practice, whereas CMT patients share common characteristics of progressive distal muscle weakness, sensory loss and deformity (Klein, 2020), they also have a clinically heterogeneous set of disorders, spanning a spectrum from mildly symptomatic forms to those resulting in severe disability. Based on median motor nerve conduction velocity (MNCV), CMT can be categorized as demyelinating type (median MNCV<38 m/s), axonal type (median MNCV>38 m/s) and intermediated type (median MNCV 25–45 m/s) (Pareyson and Marchesi, 2009). In each category, inheritance patterns may be autosomal dominant, autosomal recessive, or X-linked.

Recently, next-generation sequencing (NGS) and whole-exome sequencing (WES) have resulted in a rapid expansion of the genetic diagnosis of CMT, with over 100 genes identified and many more still to be discovered (Padilha et al., 2020). In comparison to CMT of all age groups, childhood-onset patients may present more phenotypic variability and mutation-specific manifestations (Cornett et al., 2016). In addition, it is worth noting that *de novo* cases are not rare for CMT, especially in the earlier-onset group (Landrieu and Baets, 2013; Ma et al., 2021). Therefore, owing to the clinical complexity and genetic diversity, the diagnosis of paediatric CMT is always difficult. Furthermore, previous published studies also illustrated that the distribution of subtypes varied in different geographical regions (Fridman et al., 2015). Thus, a comprehensive knowledge of the distribution of mutations within earlier-onset subgroup in different areas is important and challenging.

Paediatric CMT, with disease onset in the first 2 decades, is a group that deserves further attentions. First, the presentation of paediatric patients is quite different from that of adult patients in terms of disease severity and clinical features, as symptoms can be insidious and severely disabling. Besides, there are obvious delays in achieving motor milestones in earlier-onset patients, which may lead to a considerable socioeconomic burden (Baets et al., 2011). In addition, improved genetic diagnosis rate in paediatric patients may help facilitate clinical trials of the upcoming disease-modifying treatment. To date, studies on paediatric CMT have mainly been based on patients of European origins, with a reported diagnosis rate of 75.6%–92% (Cornett et al., 2016; Hoebeke et al., 2018; Argente-Escrig et al., 2021). However, the disease characteristics of paediatric CMT have not been extensively characterized in the Chinese population thus far, either in mutation spectrum or phenotypic analysis. Therefore, the clinical heterogeneity in patients of different origins, coupled with the expanding genetic diversity in earlier-onset CMT leads to a great need for in-depth studies on the mutational spectrum and detailed genotype-phenotype correlations in Chinese patients.

Thus, we conducted a study in a large paediatric CMT population in China, analysing the mutational distribution and clinical characteristics. In addition, we further explored the subtype frequencies and the genotype-phenotype correlations in this series, to provide some clues for clinicians in directing genetic testing strategies and selecting disease modifying therapies for early-onset CMT patients of Chinese origin.

## Materials and methods

### Participants

A cohort of early-onset (age 0–20 years) CMT patients was enrolled consecutively in this study at Peking University Third Hospital and China-Japan Friendship Hospital from 2007 to 2021. The clinical criteria used for the diagnosis of CMT are well established in the literature (Bird, 1998). For all patients, clinical features, family history and the electrophysiological data were carefully collected and recorded. Experienced neurologists who specialized in inherited neuropathies evaluated the clinical data of all patients. Disease burden were assessed by Charcot-Marie-Tooth Paediatric Scale (CMTPedS) (Burns et al., 2012). On the basis of the disease burden scores, patients were classified as having mild, moderate, or severe disease (CMTPedS of ≤14, 15–29, or ≥30, respectively). Follow-up was carried out every 6 months through telephone calls or in-person interviews. The institutional ethics committee of Peking University Third Hospital approved this study. Patients or their legal guardians provided written, informed consents to participate in this study (2019-005-02).

### Gene screening strategy and genetic analysis

Genomic DNA was collected and extracted from peripheral blood leukocytes by standard procedures according to the manufacturer’s instructions. From 2007 to 2013, the duplication/deletion mutation of *PMP22* gene was pre-excluded in all clinically suspected demyelinating CMT patients by multiplex ligation-dependent probe amplification (MLPA) according to the guidelines. Then Sanger sequencing was used to detect missense mutation of *PMP22* (NM_153321), *GJB1* (NM_0001097642) and *MPZ* (NM_000530). In patients with axonal CMT, we investigated *MFN2* (NM_014874), *GJB1, MPZ, HSPB1* (NM_001540) and *HSPB8* (NM_014365) by direct Sanger sequencing. From 2014 to 2018, a targeted NGS panel covering 165 genes associated with inherited neuropathies was introduced for all suspected cases after excluding *PMP22* duplication/deletion mutation. Since 2019, an upgraded WES (Agilent Human All Exon V6) was performed on the index patients. The samples were sequenced on the HiSeq2500 and NEXTSEQ 500 (Illumina, San Diego, CA, United States) to discover causal genes. Identified variants by NGS or WES were further validated by Sanger sequencing. All previously reported pathogenic mutations were confirmed with reference to the Human Gene Mutation Database (HGMD) (http://www.hgmd.cf.ac.uk/ac/index.php). Moreover, novel variants were interpreted and classified according to the American College of Medical Genetics and Genomics/Association for Molecular Pathology (ACMG/AMP) standards and guidelines (Richards et al., 2015). A flow chart for gene screening strategy (Supplementary Figure S1) and a list of gene panel of NGS (Supplementary Table S1) are summarized in the Supplementary Materials.

### Statistical analysis

All statistical analyses were performed using GraphPad Prism 7.0 (GraphPad Software, Inc., CA, United States) and SPSS V.23.0 software (IBM Corp., Armonk, United States). Descriptive statistics such as age at onset (AAO); electrophysiological parameters were expressed as mean ± SD (range).

## Results

### Characteristics of study participants

A total of 181 patients of Chinese descent from two clinical centres were recruited in this study. Of those patients, 41.4% (75/181) were classified as having demyelinating CMT, 47% (85/181) as having axonal CMT and 8.3% (15/181) as having intermediated CMT. In addition, six patients met the diagnostic criteria for hereditary neuropathy with liability to pressure palsies (HNPP). According to the inheritance patterns, 61 (33.7%) patients were categorized having autosomal dominant CMT, 30 (16.6%) as having autosomal recessive CMT and 12 (6.6%) as having X-linked CMT (CMTX). Furthermore, among 78 sporadic patients, 23 (12.7%) cases were identified to be of *de novo* origins. For all modes of inheritance, the median AAO was 8.3 ± 5.7 (0–19) years, with a mean diagnostic age of 14.3 ± 6.0 (0.5–19.5) years. In general, most patients first noticed symptoms such as weakness, falls and pes cavus at disease onset. Additionally, according to disease severity, *PMP22* point mutation and *MPZ* were prone to cause the most severe phenotypes. The detailed CMTPedS based on different genotypes were summarized in the Supplementary Table S2.

### Distribution of CMT subtypes

Among the different CMT subtypes, patients with demyelinating CMT presented with an earlier onset age (5.9 ± 5.5 years). Although most were associated with classic demyelinating phenotypes (55/75, 73.3%), there was considerable phenotypic heterogeneity such as prominent deep sensory disturbances (*SH3TC2, PRX*) and scoliosis (*SH3TC2, MPZ,* and *PMP22*). On electrophysiological examination, motor conduction velocity (MCV) values were uniformly decreased, with some even below 10 m/s. For the patients with axonal CMT, the AAO was higher (7.8 ± 5.6 years) than that in patients with demyelinating forms. Clinical presentation showed an absence of upper limb involvement in approximately half of the patients. Mutation-specific manifestations were also obvious, with *GARS* mutation manifesting as predominant upper limbs involvement. Moreover, 12 patients reported clinically pure motor involvement with only slight sensory impairment on electrophysiological examination. Furthermore, the disease severity also varied in axonal CMT according to different pathological mutations. In contrast, patients of intermediate CMT mainly had the classic phenotype, with a relatively benign disease course of late-onset (10.2 ± 4.5 years) and mild peripheral neuropathy (Table 1).

**TABLE 1:** Comparisons of clinical and electrophysiological data in pediatric CMT.

	CMT (*n* = 181)	Demyelinating CMT (*n* = 75)	Axonal CMT (*n* = 85)	Intermediated CMT (*n* = 15)
AAO (year)	8.3 ± 5.7	5.9 ± 5.5	7.8 ± 5.6	10.2 ± 4.5
Diagnostic age (year)	14.3 ± 6.0	12.4 ± 7.1	13.8 ± 6.3	15.6 ± 3.9
Disease duration (year)	5.9 ± 4.1	6.6 ± 4.9	6.1 ± 4.0	6.5 ± 3.7
Gender (M/F)	101/80	43/32	43/42	12/3
Clinical features				
Pes cavus (%)	66.9	73.3	65.9	66.7
Weakness in lower limbs (%)	94.5	97.3	94.1	100
Weakness in upper limbs (%)	44.2	40	43.5	60
Hypoaesthesia (%)	38.7	37.3	37.6	46.7
Deep sensory disturbance (%)	23.2	29.3	21.2	20
Scoliosis (%)	9.9	16	7.1	0
Classic phenotype (%)	61.3	73.3	48.2	66.7
Electrophysiological parameters				
Median Nerve MCV (m/s)	35.1 ± 11.8	14.6 ± 9.7	56.5 ± 3.3	29.9 ± 10.2
Median Nerve CMAP (mv)	3.9 ± 2.6	3.2 ± 2.4	5.1 ± 1.3	2.6 ± 1.8
Ulnar Nerve MCV (m/s)	36.3 ± 8.7	17.9 ± 6.8	53.3 ± 4.5	33.3 ± 6.9
Ulnar CMAP (mv)	3.6 ± 2.3	2.1 ± 1.6	4.4 ± 1.8	3.5 ± 2.1
Median Nerve SCV (m/s)	35.2 ± 14.4	21.5 ± 8.8	53.3 ± 4.5	27.2 ± 13.4
Median Nerve SNAP (μV)	2.6 ± 1.9	0.4 ± 1.1	4.6 ± 0.7	3.4 ± 2.9
Ulnar Nerve SCV (m/s)	32.9 ± 17.5	21.4 ± 8.3	22.9 ± 17.9	26.9 ± 15.9
Ulnar Nerve SNAP (μV)	2.2 ± 1.8	0.1 ± 0.4	4.7.±1.2	1.6 ± 1.4

AAO, age at onset; CMT, Charcot-Marie-Tooth disease; MCV, motor conduction velocity; CMAP, compound muscle action potential; SNAP, sensory nerve action potential; SCV, sensory nerve conduction velocity.

### Genotypes distribution characteristics

Among all 181 index patients, pathogenic mutations were identified in 123 patients, with a diagnostic rate of 68% (123/181). In all CMT subtypes, the leading causes were CMT1A/*PMP22* duplication (18.2%; 33/181), CMT2A/*MFN2* mutation (7.7%; 14/181) and CMTX1/*GJB1* mutation (6.6%; 12/181). In addition, casual mutation were identified in the following genes: *GDAP1* (5%; 9/181), *PMP22* point mutation (4.4%; 8/181), *IGHMBP2* (3.3%; 6/181), *MORC2* (3.3%; 6/181), *MPZ* (2.2%; 4/181), *SORD* (1.7%; 3/181) and *SH3TC2*(1.7%; 3/181). Furthermore, mutations in the remaining CMT-related genes (i.e., *HSPB1, PRX, BSCL2, DYNC1H1, HINT1, GARS, AARS, MARS, HK1, TRPV4, KIF5A, EGR2, FGD4, MTMR2, SPG11, NEFL, DHTKD1*) were each responsible for <1% of all CMT cases. According to the clinical subtypes, *PMP22* duplication, *MFN2* and *GJB1* were the most common causative genes in demyelinating CMT, axonal CMT and CMTX respectively.

**FIGURE 1:**
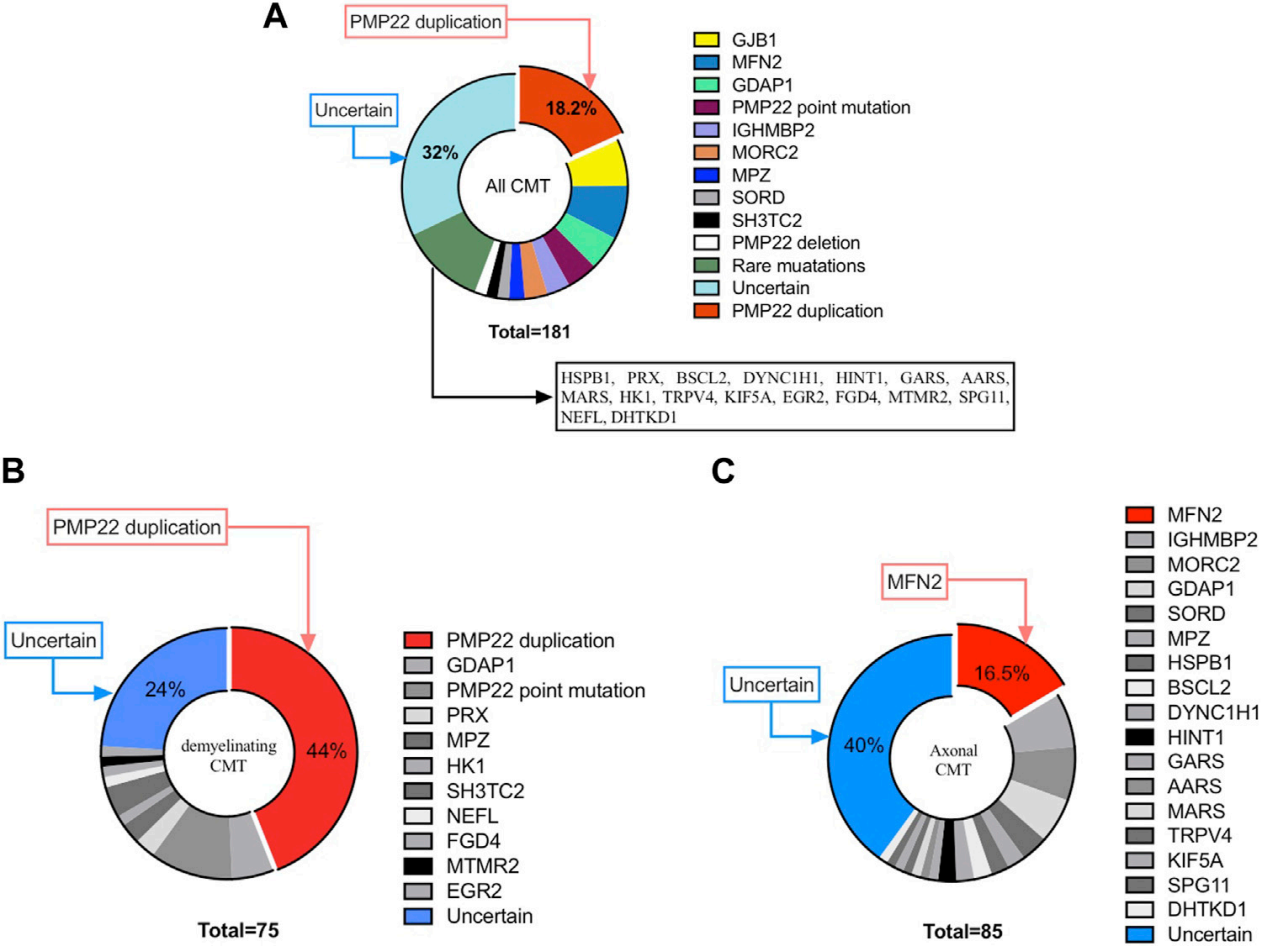
Distribution of Chinese paediatric CMT patients in our cohort. **(A)** Distribution of CMT. **(B)** Distribution of demyelinating CMT. **(C)** Distribution of axonal CMT.

Of the patients with demyelinating CMT, 76% (57/75) carried a genetic mutation, with the most frequent genetic causes being *PMP22* duplication (44%, 33/75), *PMP22* point mutation (10.7%, 8/75) and *GDAP1* mutation (5.3%, 4/75), accounting for 60% of all demyelinating CMT patients with an identified mutation. For patients with axonal CMT, 60% had a genetically confirmed diagnosis (51/85), which was lower than that for patients with the demyelinating subtype. Moreover, mutational screening showed marked genetic heterogeneity, with mutations in *MFN2* (16.5%; 14/85), *IGHMBP* (7.1%; 6/85) and *MORC2* (7.1%; 6/85) being the three most frequent causes. For intermediated CMT, specific genetic mutations were identified in 80% (12/15) of patients. Of note, up to 12.7% (23/181) of patients were found to have *de novo* mutations with the following distribution (11 mutations in *PMP22*, three in *MFN2*, three in *MPZ*, and three in *MORC2*), which was not rare. Genotype distribution details are summarized in Figure 1.

### Genotype-phenotype correlations

**FIGURE 2:**
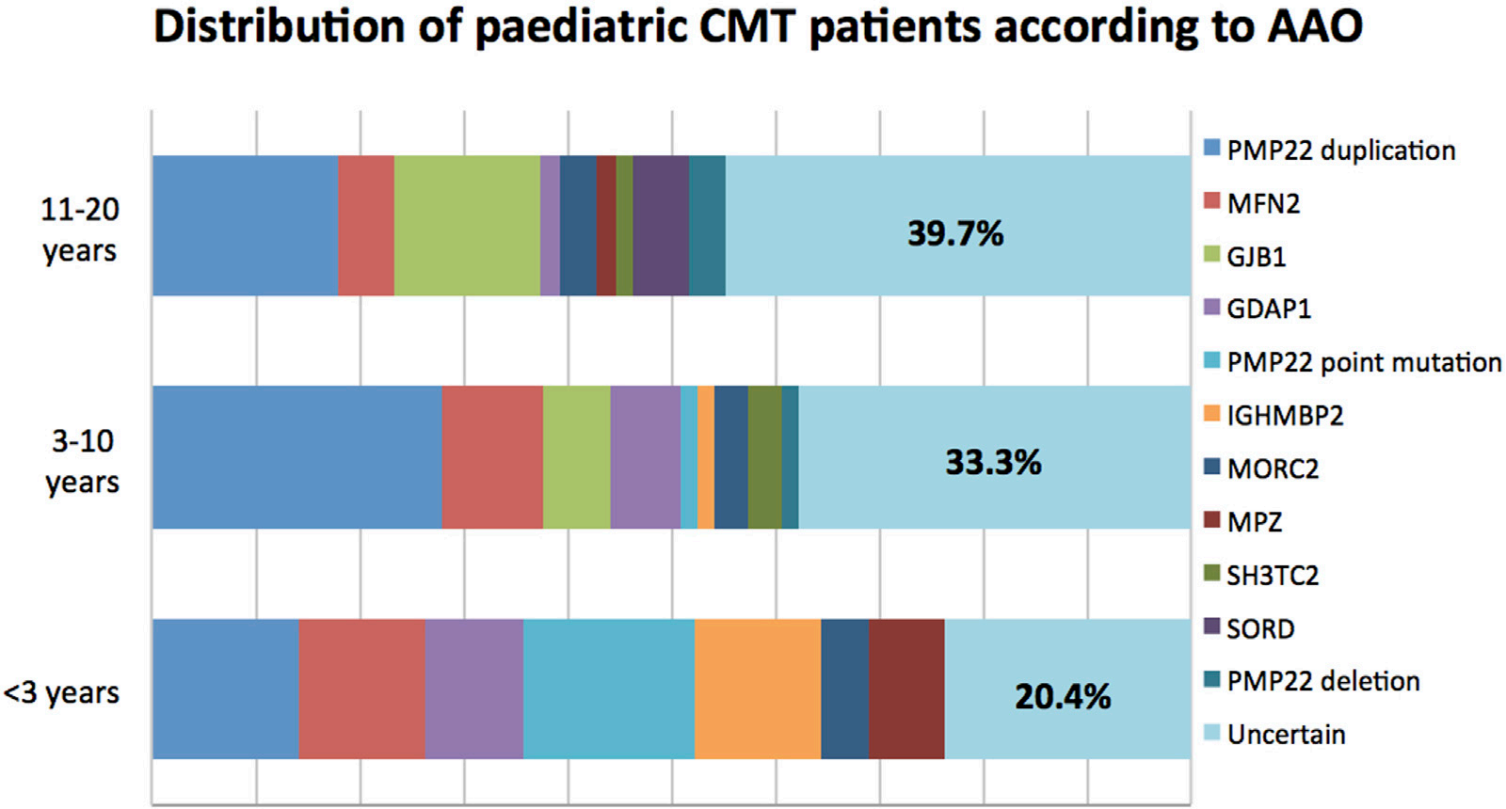
Distribution of paediatric CMT patients according to AAO.

In terms of AAO, CMT can be further categorized into infantile-onset (<3 years), childhood-onset (3–10 years) and adolescent-onset (11–20 years) subtypes. In general, childhood-onset (38.1%; 69/181) was the most common subtype, with a successful genetic diagnosis rate of 66.7% (46/69). In this group, mutations in *PMP22, MFN2, GJB1*, and *GDAP1* were the most common causal mutations. Significantly, patients with infantile-onset CMT had the highest mutation detection rate of 79.6% (39/49), among whom mutations in *PMP22, MFN2*, and *IGHMBP2* were the top three common causes of pathologies. In the adolescent-onset group, the diagnosis rate was 60.3% (38/63), with mutations in PMP22 duplication, *GJB1, MFN2,* and *SORD* being the major aetiologies. The detailed genetic distribution according to AAO is shown in Figure 2.

There were some mutation-specific manifestations among the CMT subtypes. *PMP22* point mutations were found in patients with the most severe demyelinating CMT subtypes (CMTPedS = 32.2 ± 6.5). Among these patients, 87.5% (7/8) developed the disease before the age of 3 years and 62.5% (5/8) were severely affected. Moreover, *de novo* variants at specific amino acid positions, e.g., p. S72W, p. S79P, and p. G150V caused the severe phenotypes of Dejerine-Sottas disease (DSS), which manifests as earlier onset, delayed motor development, hypotonia and profoundly slowed MCV (<15 m/s) (Agrahari et al., 2015). The second most severe paediatric CMT subtype in the cohort was caused by *MPZ* mutation. Certain *de novo* variants, such as p. R98C, p. S233Rfs*18 and the novel p. L174Rfs*66 were associated with clinical features of weakness, atrophy, deformity and motor retardation. Furthermore, patients with SH3TC2 variants also presented with a moderate to severe phenotype, which usually manifested as severe weakness, sensory ataxia and scoliosis. In particular, for patients who developed symptoms before 10 years of age, the disease seemed to be more severe than that of the other subgroups. In addition, disease progression gradually stabilized with increasing age.

With regard to disease severity, *PMP22* duplication and *MFN2* mutations were the most frequent causes of moderate phenotypes. In addition, mutations in *GJB1* and *GDAP1* were prone to cause mild phenotypes. The phenotypes of *PMP22* point mutations were confirmed to be severe clinical features, accounting for 29.4% (5/17) of the severe cases in total, followed by *MPZ* and *SH3TC2* mutations.

## Discussion

In this study, we identified genotype and phenotype distributions of paediatric CMT patients in a large Chinese cohort. We also performed an in-depth genotype-phenotype correlation study, which was the largest study focused on paediatric CMT patients in China thus far. In our findings, the mean age when parents first noticed symptoms was 8.3 ± 5.7 years, which was significantly older than that in the French study (4.1 years) (Hoebeke et al., 2018). Similarly, the diagnostic age was also significantly different between these two studies (14.3 years in the Chinese patients vs. 8.3 years in the French cohort). It should be noted that the demyelinating CMT (AAO = 5.9 ± 5.5 years) in our series only accounted for 41.4% (75/181) of all CMT compared to that of 61.3% in the French cohort, which may partly explain the differences in onset age. Besides, the longer diagnostic delay in our study may largely due to the feasibility to access to our specialized clinics rather than disease severity.

Based on clinical subtypes, demyelinating CMT manifests with earlier disease onset than other CMT subtypes. Clinically, the majority (73.3%) of demyelinating CMT patients presented with typical phenotypes, whereas axonal CMT patients varied strikingly in terms of clinical features due to specific genotypes. In addition to the classic features of progressive distal muscle weakness, CMT2A patients may present with complex phenotypes, including tremor, optic atrophy and pyramidal signs. Additionally, 14.1% of axonal CMT patients are characterized as having motor-predominant neuropathy, which is difficult to distinguish from distal hereditary motor neuropathies (dHMN) (Liu et al., 2020). For CMT1X, males were more affected than females, with a mild to moderate phenotype of adolescent-onset and intermediate slowing in electrophysiological examination, which was in consistent with previous reports (Panosyan et al., 2017).

Overall, a confirmed genetic diagnosis was achieved in 123 of 181 patients (68%), with a higher detection rate in patients with the demyelinating forms (57/75; 76%) than in those with the axonal forms (51/85; 60%). Compared among patients of all ages, the genetic confirmation rate in our paediatric cohort was similar to that reported in South China (70%) (Xie et al., 2021) and Taiwan (73.1%) (Hsu et al., 2019). However, this rate was higher than that worldwide (60.4%) (Fridman et al., 2015), suggesting that paediatric patients has a higher genetic diagnosis rate than those of all age groups. Nevertheless, this result is somewhat lower than that in other series when comparing within the paediatric groups (Cornett et al., 2016; Hoebeke et al., 2018; Argente-Escrig et al., 2021) (Table 2). To date, the largest paediatric series based on eight sites reported a diagnosis rate of 75.6% (Cornett et al., 2016). In another study from France, up to 92% of patients received a genetic diagnosis (Hoebeke et al., 2018). Compared with these studies, the reasons for our low mutation detection rate may be partly due to the small proportion of patients carrying *PMP22* duplication (18.2% of patients in our study versus 37.4%–61.3% in the aforementioned studies). It is worth noting that in other studies based on Chinese populations, CMT1A also only accounted for 19.5%–29.5% of CMT cases (Liu et al., 2020; Xie et al., 2021), with a similar rate in Japan and Korea (15%–26.3%) (Choi et al., 2004; Abe et al., 2011). The difference in the prevalence of *PMP22* duplication might be attributed to the heterogeneity among patients of different origins as well as the underestimation of mild patients in our country who did not receive detailed examination at hospitals. Therefore, this result indicates that although some asymptomatic or mild CMT1A cases may be missed in diagnosis, there is indeed distributional heterogeneity among patients of different origins.

**TABLE 2:** Gene distributions compared with previous studies.

Gene	This study (*n* = 181)	Cornett et al. (*n* = 520)	Hoebeke et al. (*n* = 75)	Argente-Escrig H. et al. (*n* = 99)
AAO (years)	0–20	3–20	0–18	0–20
*PMP22* duplication	33 (18.2)	252 (48.5)	46 (61.3)	37 (37.4)
*PMP22* point mutation	8 (4.4)	9 (1.7)	-	-
*PMP22* deletion	3 (1.7)	5 (1.0)	-	-
*GJB1*	12 (6.6)	10 (1.9)	2 (2.6)	8 (8.1)
*MFN2*	14 (7.7)	31 (6.0)	11 (14.7)	3 (3.0)
*GDAP1*	9 (5)	3 (0.6)	1 (1.3)	10 (10.1)
*IGHMBP2*	6 (3.3)	-	-	-
*MORC2*	6 (3.3)	-	-	-
*MPZ*	4 (2.2)	15 (2.9)	1 (1.3)	3 (3.0)
*SH3TC2*	3 (1.7)	13 (2.5)		2 (2.0)
*SORD*	3 (1.7)			
*HSPB1*	2 (1.1)			
*PRX*	2 (1.1)	1 (0.2)	1 (1.3)	
*BSCL2*	2 (1.1)			
*DYNC1H1*	2 (1.1)			1 (1.0)
*HINT1*	2 (1.1)			
*GARS*	1 (0.6)	4 (0.8)		
*AARS*	1 (0.6)			
*MARS*	1 (0.6)			
*TRPV4*	1 (0.6)	1 (0.2)		
*HK1*	1 (0.6)		1 (1.3)	3 (3.0)
*KIF5A*	1 (0.6)			
*EGR2*	1 (0.6)			2 (2.0)
*FGD4*	1 (0.6)	1 (0.2)		2 (2.0)
*MTMR2*	1 (0.6)	2 (0.4)		
*SPG11*	1 (0.6)			
*NEFL*	1 (0.6)	3 (0.6)		
*DHTKD1*	1 (0.6)			
Uncertain	58 (32)	127 (24.4)	6 (8.0)	20 (20.2)

AAO, age at onset.

In our current series, the most frequent disease-causing genes were *PMP22* duplication, followed by *MFN2* and *GJB1*. This distribution of genetic subtypes in our patients was similar to those reported previously in Chinese patients. A study focused on South Chinese CMT patients identified *PMP22* (19.1%–29%), *GJB1* (13.5%–13.8%) and *MFN2* (6.5%–10.1%) as the most frequent causative genes (Chen et al., 2019; Xie et al., 2021). In another study, *PMP22* (48.7%), *GJB1* (9.4%) and *MFN2* (3.3%) also ranked as the top three disease-causing genes in a Taiwanese CMT population (Hsu et al., 2019). Likewise, compared with Caucasian paediatric populations, the genetic spectrum was partly different in our cohort. Except for the remarkably lower detection rate of *PMP22* duplication in our study, mutations in *IGHMBP2, MORC2,* and *SORD*, manifesting as predominantly motor involvement, were also first discussed in pediatric CMT. In contrast, the proportions of the other frequent causative genes in our study, such as *GJB1, MFN2* and *MPZ* were similar to those reported in studies of European ancestry (Cornett et al., 2016; Hoebeke et al., 2018; Pipis et al., 2020; Argente-Escrig et al., 2021). Among rare genes, *GDAP1* was also reported in most paediatric CMT series, with a distribution of 0.6%–10.1% in Caucasian patients and 5% in our cohort.

There was marked genetic heterogeneity according to AAO in our findings. In the infantile-onset group, the diagnosis rate ranked the highest at 79.6%, with mutations in the *PMP22, MFN2,* and *IGHMBP2* genes being the three most frequent causes. As age increased, the diagnosis rate was gradually decreased, with 66.6% in the childhood-onset group and 60.3% in the adolescent-onset group. Following the three most frequent genes, the remaining common causative genes of childhood-onset CMT were *GDAP1, SH3TC2,* and *MORC2*, while *HSPB1* and *SORD* were mostly implicated in adolescent-onset CMT. In addition, disease severity was correlated with particular genotypes. Generally, earlier onset was the most predictive marker of significant disease severity for most CMT subtypes, while for CMT1A, the disease worsened consistently throughout childhood and adolescence, which was in line with previous studies (Cornett et al., 2016). For all subtypes, symptoms related to disease severity including decreased hand dexterity and weakness in ankle dorsiflexion. In comparison to patients with other genotypes, patients with *PMP22* point mutation developed a severe phenotype with significant disability, accounting for 29.4% of the severe cases in total. In the CMT2A subgroup, patients with an AAO before 10 years tended to have more severe disease than those in the late-onset subgroup.

The most common phenotypes for infantile-onset patients were DSS in our findings. Genetically, most cases are caused by a dominant or *de novo* mutation in the *PMP22, MPZ* or *EGR2* genes (Gargaun et al., 2016; Grosz et al., 2019; Yoshimura et al., 2019). In addition to *PMP22* mutations, variants in rare genes, e.g., *EGR2, PRX*, and *FGD4* were found more frequently due to the introduction of NGS and WES, which was consistent with previous studies (Yoshimura et al., 2019).

Interestingly, the presence of *de novo* variants was identified in 12.7% of all patients and thus was not rare. Certain *PMP22* variants, such as p. S72W, p. S79P and p. G150V, were mainly observed to occur *de novo*. In addition, patients carrying *de novo* variants of *MPZ* (p. R98C, p. S233Rfs*18 and p. L174Rfs*66) were also uncommon, with a severe DSS phenotype of motor retardation, weakness and foot deformity. In previous studies*, de novo* variants for *MFN2* were not rare. In some series of CMT2A patients, a frequency of *de novo MFN2* variants has been reported of 14.4%–35.0% (Ma et al., 2021; Verhoeven et al., 2006; Kijima et al., 2005). In pediatric group, the frequency may be higher, with up to 45.5% (5/11) being reported of *de novo* in a French CMT2A cohort (Hoebeke et al., 2018). In literature, *de novo* variants are common disease mechanism in some childhood onset inherited diseases; it is associated with a reduced life expectancy and reduced reproductive fitness. As *de novo* variants are usually too deleterious to be passed on in evolution, it is more common in pediatric patients rather than other age groups (Mohiuddin et al., 2022). Compared with European paediatric CMT patients, in whom *de novo* variants were identified in only 6%–6.5% (Yoshimura et al., 2019; Argente-Escrig et al., 2021), the frequency in our cohort was to some extent higher. Except for the possible geographic differences, the reason that less awareness of disease might deceive mild non-*de novo* individuals to access to our specialized clinics may also contribute to the lower frequency in our cohort. Considering that the prevalence of *de novo* variants was highest in early-onset and severe cases, genetic screening should be performed in patients with early-onset and severe peripheral neuropathy regardless of family history.

In our cohort, variants in *IGHMBP2, MORC2,* and *SORD* accounted for a certain proportion of paediatric CMT patients, which was not reported in previous studies from Western countries. Recently, the *SORD* gene has been considered one of the most frequent causative genes for autosomal recessive axonal CMT or dHMN (Cortese et al., 2020), which share a phenotype of motor-predominant peripheral neuropathy. In this study, we identified three patients carrying either a homozygous or a compound heterozygous c.757delG (p. Ala253GlnfsTer27) variant, which was consistent with the literature (Liu et al., 2021). However, *MORC2* variants are clinically heterogeneous with phenotypes that can be characterized by congenital or early-onset spinal muscular atrophy like or pure motor axonal neuropathy (Sevilla et al., 2016). Therefore, genetic screening according to the clinical phenotype should be conducted in paediatric patients in whom CMT is suspected especially for autosomal recessive inheritance or newly discovered genes.

In summary, this study represented a major effort to investigate paediatric CMT characteristics in a Chinese population. In-depth analysis highlighted the relatively lower detection rate of *PMP22* duplication and higher frequency of *de novo* variants among paediatric patients in this specific geographic region. In addition, we illustrated genetic heterogeneity according to AAO, disease severity and clinical features. Indeed, since patients were recruited from two clinic centres, the results of the study could not represent the whole Chinese pediatric population. In the future, longitudinal studies and multi-centre studies would yield more information and analytical results.

## Data Availability

The raw data supporting the conclusion of this article will be made available by the authors, without undue reservation.
