# Supplementary figures and images for: Clinical and mutational spectrum of paediatric Charcot-Marie-Tooth disease in a large cohort of Chinese patients
Source: Front Genet. 2023 Jul 13;14:1188361. doi: 10.3389/fgene.2023.1188361 (PMC10381926; doi:10.3389/fgene.2023.1188361)

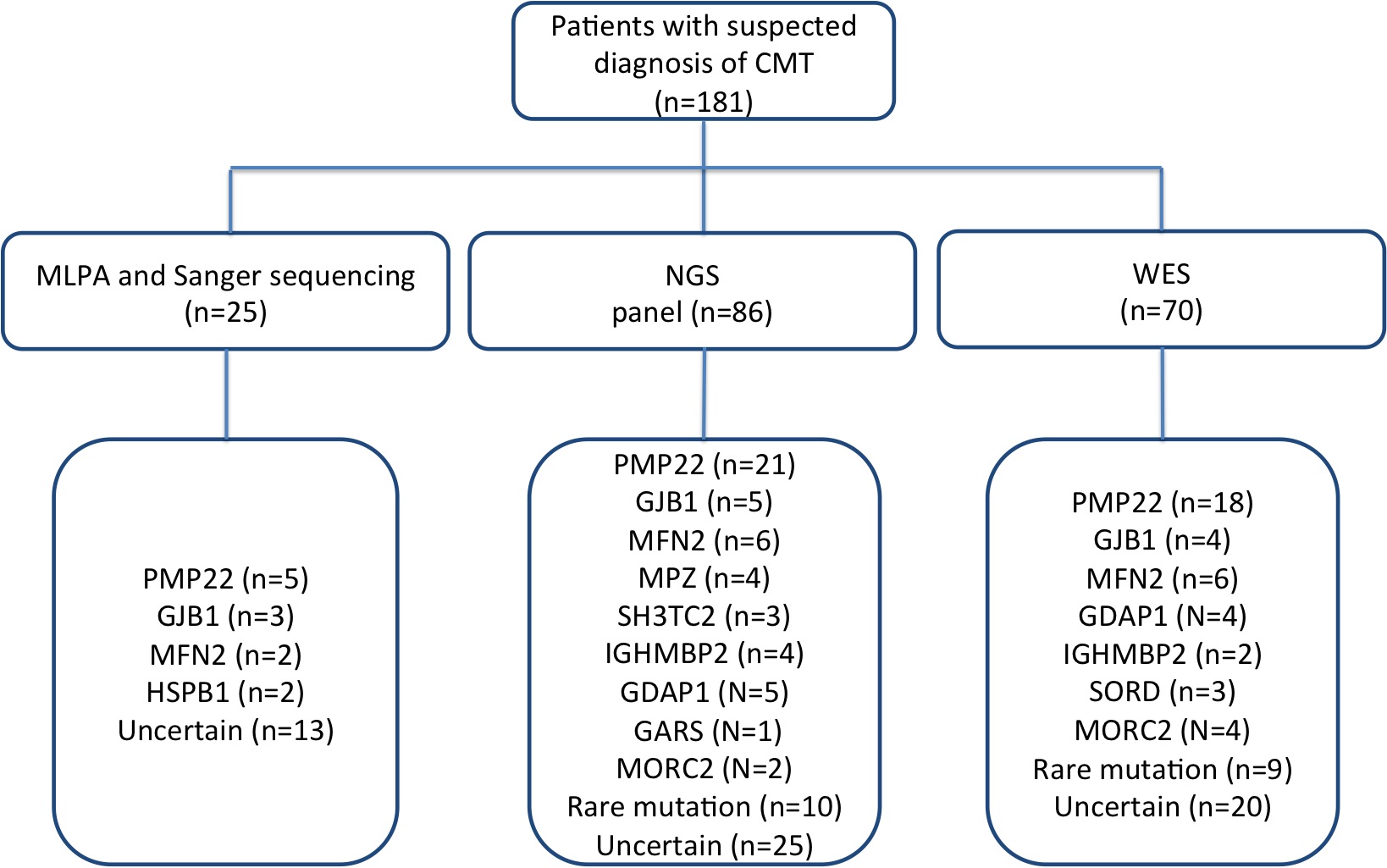

Supplement: Supplementary file 1 [file Image1.JPEG]
