# Supplementary material for: Clinical and mutational spectrum of paediatric Charcot-Marie-Tooth disease in a large cohort of Chinese patients
Source: Front Genet. 2023 Jul 13;14:1188361. doi: 10.3389/fgene.2023.1188361 (PMC10381926; doi:10.3389/fgene.2023.1188361)
Supplement: Supplementary file 2 [file DataSheet1.docx]

**SUPPLEMENTARY TABLE S1. The list of a targeted NGS gene panel for CMT in our cohort.**

| *PMP22* | *FIG4* | *EGR2* | *DNAJB2* | *AIFM1* | *DNM2* | *DNMT1* | *WNK1* | *PMM2* |
| --- | --- | --- | --- | --- | --- | --- | --- | --- |
| *MPZ* | *SURF1* | *PRX* | *MME* | *PRPS1* | *YARS* | *FBXO38* | *SCN9A* | *RBM28* |
| *LITAF* | *GJB1* | *MFN2* | *MARS* | *Xp22.2* | *DYNC1H1* | *MAPT* | *IKBKAP* | *RNF170* |
| *FGD4* | *HOXD10* | *DCTN1* | *NAGLU* | *Xq26* | *INF2* | *SLC5A7* | *SCN11A* | *SCP2* |
| *PTRH2* | *FBLN5* | *KIF1B* | *HARS* | *GAN* | *GNB4* | *VRK1* | *DST* | *MMACHC* |
| *NEFL* | *CTDP1* | *RAB7A* | *VCP* | *DCAF8* | *DHTKD1* | *PLP1* | *CCT5* | *SNAP29* |
| *KARS* | *ERCC8* | *TRPV4* | *MORC2* | *KIF5A* | *HK1* | *SPAST* | *AAAS* | *CYP27A1* |
| *ABCD1* | *ASAH1* | *GARS* | *NEFH* | *IFRD1* | *ARHGEF10* | *PDK3* | *BICD2* | *HSD17B4* |
| *PMP2* | *GALC* | *SIL1* | *MTATP6* | *BSCL2* | *NPC1* | *LAS1L* | *CAV1* | *GLA* |
| *PRNP* | *ARSA* | *HSPB1* | *TFG* | *TUBB3* | *FAM126A* | *SETX* | *CUL4B* | *FRDA* |
| *GDAP1* | *PHYH* | *12q12-q13.3* | *DGAT2* | *KIF1A* | *PLEKHG5* | *SEPT9* | *DHH* | *AGL* |
| *MTMR2* | *PEX7* | *LRSAM1* | *LMNA* | *SLC12A6* | *COX6A1* | *VAPB* | *EXOSC3* | *C10orf2* |
| *SBF2* | *PEX1* | *CD59* | *MED25* | *SCYL1* | *DRP2* | *SPTLC1* | *FLVCR1* | *MPV17* |
| *SBF1* | *ABHD12* | *MTP* | *8q13-q23* | *TDP1* | *HSPB3* | *SPTLC2* | *GBE1* | *NALCN* |
| *SH3TC2* | *DNAJC3* | *HSPB8* | *ATL1* | *PLA2G6* | *SMN1* | *FAM134B* | *GRN* | *RPIA* |
| *NDRG1* | *SOX10* | *SPG11* | *TRIM2* | *REEP1* | *ATP7A* | *NTRK1* | *HEXA* | *FAH* |
| *SLC25A46* | *GJB3* | *AARS* | *IGHMBP2* | *HINT1* | *10q24.1-q25.1* | *NGF* | *HNRNPA2B1* | *ERCC6* |
| *ABCA1* | *DDHD1* | *HFE* | *LSM11* | *NARS1* | *NARS2* | *NPC2* | *AMACR* | *NGLY1* |
| *NEFH* | *POLG* | *TREX1* |  |  |  |  |  |  |

NGS: next generation sequencing

**SUPPLEMENTARY TABLE S2. The detailed CMTPedS based on different genotypes.**

| **Gene** | **AAO** | **CMTPedS** | **Clinical features** |
| --- | --- | --- | --- |
| ***PMP22* point mutation (n=8)** | 4.0±5.6 | 32.2±6.5 | 4/8 DSS |
| ***MPZ* (n=4)** | 3.4±5.2 | 30.3±5.3 | 3/4 DSS |
| ***SH3TC2* (n=3)** | 10.3±3.5 | 23.6±2.1 | walking difficulties, no cranial involvement; 1/3 scoliosis and proprioceptive ataxia |
| ***IGHMBP2* (n=6)** | 2.5±3.1 | 20.5±3.1 | axl CMT |
| ***MFN2* (n=14)** | 6.3±5.1 | 19.5±5.1 | axonal CMT |
| ***MORC2* (n=6)** | 8.2±6.1 | 19.0±4.1 | predominantly motor involvement |
| ***PMP22* duplication (n=33)** | 8.2±4.9 | 18.7±5.2 | CMT1A |
| ***GDAP1* (n=9)** | 4.8±4.2 | 18.1±5.4 | axonal or demyelinating CMT |
| ***SORD* (n=3)** | 15.3±0.6 | 18.0±4.8 | predominantly motor involvement |
| ***GJB1* (n=12)** | 12.1±3.1 | 16.9±3.1 | CMTX |
| ***PMP22* deletion (n=3)** | 9.0±7.5 | 7.0±1.7 | HNPP |
